# Mesenchymal Stem Cell‐Derived Extracellular Vesicles as Mediators of Anti‐Inflammatory Effects: Endorsement of Macrophage Polarization

**DOI:** 10.1002/sctm.16-0363

**Published:** 2017-01-31

**Authors:** Claudia Lo Sicco, Daniele Reverberi, Carolina Balbi, Valentina Ulivi, Elisa Principi, Luisa Pascucci, Pamela Becherini, Maria Carla Bosco, Luigi Varesio, Chiara Franzin, Michela Pozzobon, Ranieri Cancedda, Roberta Tasso

**Affiliations:** ^1^ Department of Experimental MedicineUniversity of GenovaGenovaItaly; ^2^ U.O. Regenerative MedicineIRCCS AOU San Martino‐IST, National Cancer Research InstituteGenovaItaly; ^3^ U.O. Molecular PathologyIRCCS AOU San Martino‐IST, National Cancer Research InstituteGenovaItaly; ^4^ Department of Veterinary MedicineUniversity of PerugiaPerugiaItaly; ^5^ Molecular Biology LaboratoryIstituto Giannina GasliniGenovaItaly; ^6^ Department of Internal MedicineUniversity of GenovaGenovaItaly; ^7^ Stem Cells and Regenerative Medicine LabFondazione Istituto di Ricerca Pediatrica Città della SperanzaPadovaItaly; ^8^ Department of Women and Children HealthUniversity of PadovaPadovaItaly

**Keywords:** Extracellular vesicles, Mesenchymal stem cells, Inflammation, Macrophages, Cell hypoxia, Regenerative medicine

## Abstract

Mesenchymal Stem Cells (MSCs) are effective therapeutic agents enhancing the repair of injured tissues mostly through their paracrine activity. Increasing evidences show that besides the secretion of soluble molecules, the release of extracellular vesicles (EVs) represents an alternative mechanism adopted by MSCs. Since macrophages are essential contributors toward the resolution of inflammation, which has emerged as a finely orchestrated process, the aim of the present study was to carry out a detailed characterization of EVs released by human adipose derived‐MSCs to investigate their involvement as modulators of MSC anti‐inflammatory effects inducing macrophage polarization. The EV‐isolation method was based on repeated ultracentrifugations of the medium conditioned by MSC exposed to normoxic or hypoxic conditions (EV^Normo^ and EV^Hypo^). Both types of EVs were efficiently internalized by responding bone marrow‐derived macrophages, eliciting their switch from a M1 to a M2 phenotype. In vivo, following cardiotoxin‐induced skeletal muscle damage, EV^Normo^ and EV^Hypo^ interacted with macrophages recruited during the initial inflammatory response. In injured and EV‐treated muscles, a downregulation of IL6 and the early marker of innate and classical activation Nos2 were concurrent to a significant upregulation of Arg1 and Ym1, late markers of alternative activation, as well as an increased percentage of infiltrating CD206^pos^ cells. These effects, accompanied by an accelerated expression of the myogenic markers *Pax7*, *MyoD*, and *eMyhc*, were even greater following EV^Hypo^ administration. Collectively, these data indicate that MSC‐EVs possess effective anti‐inflammatory properties, making them potential therapeutic agents more handy and safe than MSCs. stem cells translational medicine *2017* Stem Cells Translational Medicine *2017;6:1018–1028*

**Significance Statement**

The rational control of inflammation, a universal response to injury, is a good candidate for triggering tissue repair. Mesenchymal stem cell (MSC) paracrine activity favors tissue repair modulating inflammation‐associated immune cells. An alternative approach to regulate the inflammatory response is here proposed. This strategy relies on the use of extracellular vesicles (EVs) derived from human adipose tissue‐derived MSCs as mediators of the anti‐inflammatory effects, switching macrophages into an alternative activation profile. Despite the remaining challenges, these results highlight the importance of EVs as a handy cell‐free approach guiding regenerative processes.

## Introduction

Tissue repair, sometimes called healing, refers to the restoration of tissue architecture and function after an injury 1. It is a multistep, dynamic process and consists of three consecutive and overlapping stages: inflammation, new tissue formation, and remodeling 2. The transition from one stage to another is controlled and regulated by cell‐released mediators, which are common to most regenerating tissues, with exception of some specialized ones, such as liver and skeletal tissues, that possess distinctive forms of regeneration and follow separate pathways 3. There is an increasing evidence that the inflammatory microenvironment resulting from the initial cell interactions dictates how the healing process will proceed 4. In particular, innate immune cells, such as macrophages, lead the inflammatory cascade reaction guiding revascularization and repair at injury sites 5, 6. Diversity and plasticity are distinctive characteristics of macrophages. Classical M1 and alternative M2 activated macrophages represent two extremes of a dynamic state of activation. M1 macrophages exhibit potent antimicrobial properties, high capacity to present antigen, and consequent activation of Th1 responses. Conversely, M2 macrophages possess the capacity to facilitate tissue repair and regeneration 7.

The contribution of mesenchymal stem cells (MSCs) in tissue repair has been addressed in a variety of disease models 8, 9. Contextually, their efficacy in the functional improvement of injured tissues was mostly related to a paracrine effect rather than a direct engraftment and differentiation 10, 11, 12. We have recently demonstrated that in an inflammatory environment as the one generated during the early phases of the wound healing process, MSC paracrine activity was significantly modulated promoting a functional switch of macrophages from a pro‐ to an anti‐inflammatory state, thus corroborating evidences showing that the mobilization of innate immune cells mediates the activation of regenerative processes 10, 13.

Among the factors responsible for the paracrine effects of MSCs, extracellular vesicles (EVs) have been recently described as new players in cell‐to‐cell communication by serving as vehicles for transfer between cells of membrane and cytosolic proteins, lipids, and genetic information 14, 15. EVs are defined as a mixed population of membrane‐surrounded structures with overlapping composition, density, and sizes, including exosomes, ectosomes, microvesicle particles, and apoptotic bodies in accordance with the recommendations of the International Society for Extracellular Vesicles (ISEV) 16.

Recent studies demonstrated that EVs represent physiologically relevant and powerful components of the MSC secretome, playing important roles in local induction of tissue regeneration 8, 12, 17. In the present study, we focused on the detailed characterization of EVs released by human adipose tissue‐MSCs to evaluate if the crosstalk between MSCs and cells of the innate immunity could be carried out by secreted EVs and if these interactions occur also in a regenerative microenvironment as the one generated following skeletal muscle damage. To mimic the typical environment established during tissue injury, EVs were isolated from the conditioned medium of MSCs harvested under both normoxic and hypoxic culture conditions (EV^Normo^ and EV^Hypo^, respectively). We here report that both types of vesicles acted as mediators of the dynamic interplay between MSCs and cells of the innate immunity in vitro and in vivo. EVs effectively triggered the macrophage proliferation and polarization from a M1 to a M2 phenotype. Of note, the hypoxic preconditioning induced an intensified release of EVs enriched with miRNAs involved in different stages of the healing process. Taking advantage of a cardiotoxin (CTX)‐induced skeletal muscle injury model, we confirmed a potent EV‐mediated anti‐inflammatory effect, through the significant downregulation of the inflammatory cytokine *IL6* accompanied by the concomitant upregulation of *IL10*. At the same time we observed also a downregulation of the M1 marker *Nos2* and an increased expression of the putative M2 markers *Arg1* and Ym1, together with an increased percentage of CD206^pos^ cells infiltrating damaged and EV‐treated muscles.

## Materials and Methods

### Mice

C57Bl/6 (MHC H2b haplotype) male mice between 3 and 5 month old were used. All mice were bred and maintained at the Animal Facility of “IRCCS Azienda Ospedaliera Universitaria San Martino – IST, Istituto Nazionale per la Ricerca sul Cancro.” All animal procedures were approved by the Local Ethical Committee and performed in accordance with the national current regulations regarding the protection of animals used for scientific purpose (D. Lgs. 4 Marzo 2014, n. 26, legislative transposition of Directive 2010/63/EU of the European Parliament and of the Council of 22 September 2010 on the protection of animals used for scientific purposes).

### Adipose Tissue‐Derived MSCs Isolation and Culture

Subcutaneous adipose tissue in the form of liposuction aspirates was obtained from human healthy donors (*n* = 18) during routine lipoaspiration after informed consent. Protocol and procedures were approved by the local ethical committee. For more details regarding MSC isolation and characterization, see Supporting Information Materials and Methods.

### Bone Marrow‐Derived Macrophage Isolation and Culture

Bone marrow (BM)‐derived macrophages (Mϕ) were isolated from C57Bl/6 mice by flushing the BM with 5 ml of Phosphate Buffered Saline (PBS), as previously described 10. Each primary culture was obtained from the BM of 3 mice, and a total of 6 primary cultures were used. Details are in Supporting Information Materials and Methods.

### Preparation of MSC Conditioned Media and EV Isolation

EVs were isolated from the conditioned media derived from human MSCs. When cells reached a confluence of 80%, extensive washes in PBS were performed to remove any possible residue of FBS. The cells were transferred in EV‐isolation medium (serum‐free Dulbecco‐Modified Eagle Medium (D‐MEM) not supplemented with Fibroblast Growth Factor‐2) and the culture split into two subcultures maintained for 48 hours under normoxic (20% O_2_) and hypoxic (1% O_2_) condition, respectively. EVs were isolated from normoxic‐ and hypoxic‐conditioned media (EV^Normo^ and EV^Hypo^) by differential centrifugation at 300*g* for 10 minutes, 2,000*g* for 20 minutes, 10,000*g* for 30 minutes at 4°C to eliminate cells and debris. Obtained supernatants were depleted of residual floating cells and cell debris by filtration with 0.22 μm filter units (Merck Millipore Ltd, Vimodrone, MI, Italy), followed by two consecutive steps of ultracentrifugation at 100,000*g* for 90 minutes, including a washing step in PBS, to precipitate EVs. A Beckman Coulter ultracentrifuge (Beckman Coulter Optima L‐90K ultracentrifuge; Beckman Coulter, Fullerton, CA) was used with swinging bucket rotors type SW28 and SW41Ti. EVs were collected in 100 μl of filtered PBS and used immediately after isolation.

### Transmission Electron Microscopy

The morphological evaluations of isolated EV^Normo^ and EV^Hypo^, and corresponding MSC monolayers were performed by transmission electron microscopy (TEM). For details, see Supporting Information Materials and Methods.

### Protein Quantification and Immunoblot Analysis

Protein contents of isolated EVs were measured using a BCA protein assay kit (Thermo Scientific Pierce, Rockford, IL) following manufacturer's instructions. Sample preparation for immunoblot analysis is described in Supporting Information Materials and Methods.

### Cell Viability and BrdU Cell Proliferation Assay

3 × 10^4^ Mϕ in serum free medium were plated in 96‐well plates for 24 hours in the presence or absence of either EV^Normo^ or EV^Hypo^. Cell proliferation was measured with the use of the Cell Proliferation Enzyme‐linked immunosorbent assay (ELISA), Bromodeoxyuridine (BrdU) (Roche Mannheim, Germany), according to the manufacturer's instructions. Five independent experiments were performed.

### In Vivo Angiogenic Assay

The in vivo angiogenic assay is described in Supporting Information Materials and Methods.

### Flow Cytometry Analysis

At least nine independent preparations of both EV^Normo^ and EV^Hypo^ were stained with 10 μM Cell Trace (Molecular probes) in combination with the mouse anti‐human monoclonal antibody (mAb) CD63 (Clone: H5C6) (BD Pharmingen) or the anti‐human mAb CD105 (Clone: SN6) (eBioscience). A set of microsphere suspensions (1, 4 μm) (Molecular Probes) was used as size reference. An unstained sample was acquired to detect the sample auto‐fluorescence and set the photomultiplier for all the three used channels; fluorescent spill‐over was controlled by spectral overlap adjustment, acquiring single‐color stained tubes. Forward and side scatter channels (FSC and SSC) were used on a logarithmic scale visualized in bi‐exponential mode. The FSC and SSC photomultipliers were set using background noise as the lower optical limit, acquiring a sample of sterile PBS tube. The threshold, set on the FSC channel, was regulated to reduce the noise progressively, allocating dots in low left corner of plot, in order to clearly detect EVs. Details about the absolute count of EVs, the immunophenotype of Mϕ cultured in presence/absence of EVs and the immunophenotype of Mϕ infiltrating the injured *tibialis anterior* (TA) muscles are reported in Supporting Information Materials and Methods.

### RNA Extraction

RNA extraction procedure for both EV pellet and TA muscles is described in Supporting Information Materials and Methods.

### microRNA Profiling

The miRNA fraction of each sample was subjected to stem‐loop RT‐qPCR amplification, as described in Supporting Information Materials and Methods.

### Quantitative Real‐Time PCR

To validate the RNA sequencing data, we performed a qPCR analysis of miR‐199a‐3p, miR‐126, miR‐223, and miR‐146b. Each microRNA was tested on three independent preparations of both EV^Normo^ and EV^Hypo^, and three independent experiments were performed. The miRNA‐specific miScript Primer Assays were purchased from QIAGEN (MS00007602 for miR‐199a‐3p, MS00003430 for miR‐126, MS00003871 for miR‐223, and MS00003542 for miR‐146b). Details reported in Supporting Information Materials and Methods.

Details about the quantification of *IL‐6, IL‐10, Nos2, Arg1, Ym1, MCP1, eMyhc, Pax7,* and *MyoD* mRNAs in TA muscles of CTX and EVs injected mice are described in Supporting Information Materials and Methods.

### Labeling and Internalization of EVs

EV^Normo^ and EV^Hypo^ (derived from three different MSC cultures) were labeled using PKH67 membrane‐binding fluorescent labels according to manufacturer's recommendations (Sigma‐Aldrich, Allentown, PA).

Three independent primary cultures of Mϕ seeded on glass slides placed in 24‐well plates were incubated at 37°C with labeled EVs at a concentration of 1 μg EVs/10,000 cells. Uptake was stopped after 3 hours by washing and fixation in 4% paraformaldehyde for 20 minutes.

### Immunofluorescence Analysis

Immunofluorescence analysis performed on Mϕ is included in Supporting Information Materials and Methods.

### Mouse Model of Cardiotoxin‐Induced Muscle Injury

Eight‐week‐old male C57BL/6 mice (six per group) were anesthetized with isoflurane. Twenty microliter of 10 mM cardiotoxin (CTX) (Sigma) in PBS were intramuscularly administered into the TA muscle of both legs. One microgram of EVs (diluted in 20 μl PBS) derived from normoxic and hypoxic MSCs were injected into the right and left TA muscles, respectively. Control mice were treated with 20 μl of vehicle solution. EVs or vehicle solution were injected 2 hours postadministration of CTX and a boost of EVs was done 4 days after muscle injury. Mice were sacrificed after 1, 2, and 7 days post lesion induction and the harvested TA muscles were snap‐frozen in liquid nitrogen before further RNA extraction processing.

### Histology and Morphometric Analysis

The histological analysis of differentially‐treated muscle tissues is described in Supporting Information Materials and Methods.

### Statistical Analysis

All results were expressed as mean ± SD or as mean ± SEM from at least three independent experiments. Statistical comparisons between two groups were performed using an unpaired two‐tailed Student's *t* test. Differences among multiple groups were statistically analyzed employing One‐way ANOVA and Tukey's multiple comparisons test. A *p* value below.05 was considered to be statistically significant. All statistical analyses were performed using GraphPad Prism Version 6.0a (GraphPad Software, La Jolla, CA).

## Results

### Hypoxic Conditioning of MSCs Enhances the Release of EVs Endowed With Angiogenic Potential

**Figure 1:**
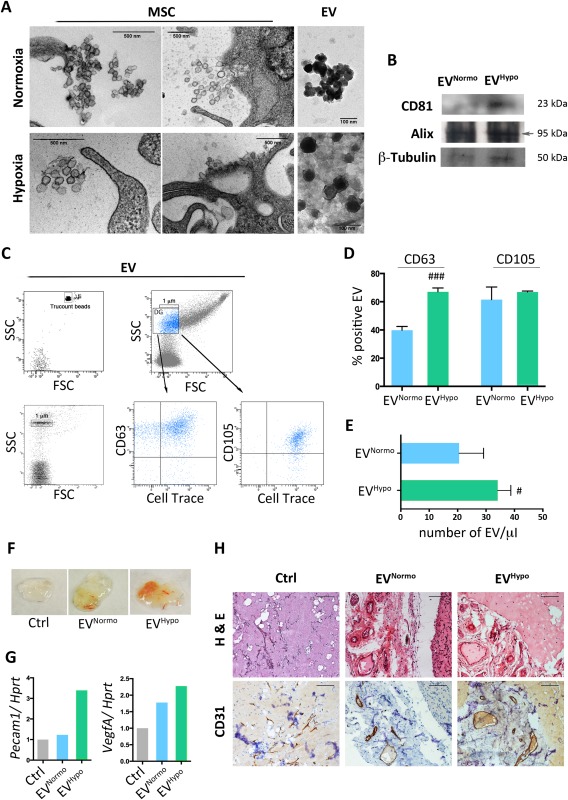
MSC‐derived EV characterization. **(A)**: Transmission electron microscopy analysis of MSC cultured for 48 hours in normoxic and hypoxic conditions and corresponding isolated EV^Normo^ and EV^Hypo^. Scale bars: MSC panels = 500 nm; EV panels = 100 nm. **(B)**: Representative Western blot analysis for EV specific markers (CD81 and Alix) and for β‐Tubulin. **(C)**: Flow cytometry strategy adopted to characterize EV^Normo^ and EV^Hypo^. Vesicles were stained by the lipophilic dye Cell Trace, the vesicular marker CD63 and the MSC marker CD105. **(D)**: Histogram showing the percentage of Cell Trace^pos^ EVs coexpressing CD63 or CD105. Data are presented as mean ± SD. ###, *p* = .0003 (unpaired Student's *t* test). (**E)**: Histogram showing the absolute number of Cell Trace^pos^ EVs quantified trough Trucount fluorescent beads. Data are presented as mean ± SD. #, *p* = .0318 (unpaired Student's *t* test). **(F)**: Representative photo of Matrigel plugs in combination with PBS (Ctrl), 5 μg of EV^Normo^ or 5 μg of EV^Hypo^, recovered 3 weeks after their in vivo injection. **(G)**: Histograms summarizing densitometric analysis of PCR products normalized to the housekeeping gene *Hprt*. Pecam1; VegfA. **(H)**: Representative histology of Matrigel plug sections stained for hematoxylin and eosin (upper panels) and for the endothelial cell marker CD31 (lower panels). Magnification ×40. Scale bar = 100 μm. Abbreviations: EVs, extracellular vesicles; FSC, forward scatter channels; *Hprt,* hypoxanthine guanine phosphoribosyltransferase; MSCs, mesenchymal stem cells; Pecam1, platelet and endothelial cell adhesion molecule 1; SSC, side scatter channels; VegfA, vascular endothelial growth factor A.

The cargo and function of EVs depend on their cells of origin, suggesting that intercellular communication through vesicles is a dynamic system, adapting its message depending on the conditions of the producing cells 18. Changes in oxygen concentrations affect many of the distinctive characteristics of stem and progenitor cells 19. On this basis, we evaluated whether hypoxic conditioning of human adipose tissue‐derived MSCs could influence their EV secretion. Confluent primary MSC cultures fulfilling the minimal criteria proposed by the International Society for Cellular Therapy 20 (Supporting Information Fig. 1A) were maintained for 48 hours in serum‐free medium in a normoxic or hypoxic environment. After the starvation period, more than 85% of MSCs resulted viable in both culture conditions (Supporting Information Fig. 1B). As expected, MSCs cultured in hypoxic conditions had a higher level of HIF‐1α expression than those cultured in normoxic conditions (Supporting Information Fig. 1B). After 48 hours of medium conditioning, isolated EV^Normo^ and EV^Hypo^, and corresponding MSC monolayers (MSC^Normo^ and MSC^Hypo^) were analyzed by TEM. TEM revealed the presence of larger shedding vesicles (microvesicles) as well as several multivesicular bodies (MVBs) containing exosomes within the cell cytoplasm in both culture conditions, indicating the release of a mixed population of EVs (Fig. 1A). In both samples, EVs appeared with a round‐shape morphology, mainly isolated or less frequently aggregated in small groups. They showed a diameter ranging from 40 to 250 nm suggesting that the separation procedure selected a population of nano‐scaled vesicles referable mostly but not only to exosomes. No morphological differences between EV^Normo^ and EV^Hypo^ were observed with regard to their size, shape, or electrondensity (Fig. 1A). In order to characterize isolated EVs, immunoblot and flow cytometry analysis were performed. Western blot analysis revealed that both EV^Normo^ and EV^Hypo^ express the specific vesicular protein CD81, member of the tetraspanin family, and Alix, that is involved in MVB formation (Fig. 1B). EV^Normo^ and EV^Hypo^ were further characterized taking advantage of a multiparametric flow cytometry approach. To separate true events from background noise, EVs were defined as events that were included in the dimensional gate of 1 μm, which was established according to a well‐defined light scatter profile of beads with absolute size (Fig. 1C). EVs were targeted with the Cell Trace labeling, in order to consider only intact membrane structures, along with either the mesenchymal marker CD105 or the vesicular marker CD63. Both types of Cell Trace labeled‐EVs expressed the CD105 and CD63 antigens, but the percentage of EVs co‐expressing CD63 was significantly higher in the hypoxic condition compared to the normoxic one (*p* < .01) (Fig. 1D). The absolute quantification of EV^Normo^ and EV^Hypo^ was determined by comparing their events to a known number of fluorescent bead events (Trucount beads, Fig. 1C). The hypoxic conditioning induced a significantly increased release of EVs when compared to the normoxic condition (*p* = .0318) (Fig. 1E).

The observations that the regenerative properties mediated by MSCs, including the ability to stimulate angiogenesis, are mediated by EV secretion, and that hypoxia is a factor that favors the accumulation of pro‐angiogenic molecules 21, led us to explore the angiogenic potency of MSC‐EVs in vivo by performing the Matrigel plug assay. After 3 weeks of implantation, we observed that EV^Normo^ and EV^Hypo^ induced the formation of vessel‐like endothelial structures (Fig. 1F). Matrigel plugs in presence of both types of vesicles were enriched in angiogenic molecules, such as *Pecam1* and *VegfA* when compared with control empty plugs (Fig. 1G). The presence of vessels along the periphery of the plugs was confirmed in all the experimental conditions by hematoxylin and eosin and CD31 immunostaining (Fig. 1H). Noteworthy, in EV ^Hypo^‐treated plugs, a higher expression of *Pecam1* and *VegfA* and an increased density of vessels with a larger diameter were detectable (Fig. 1G, 1H).

### EVs Secreted Under Hypoxia Express miRNAs Actively Involved in Different Stages of the Healing Process

**Figure 2:**
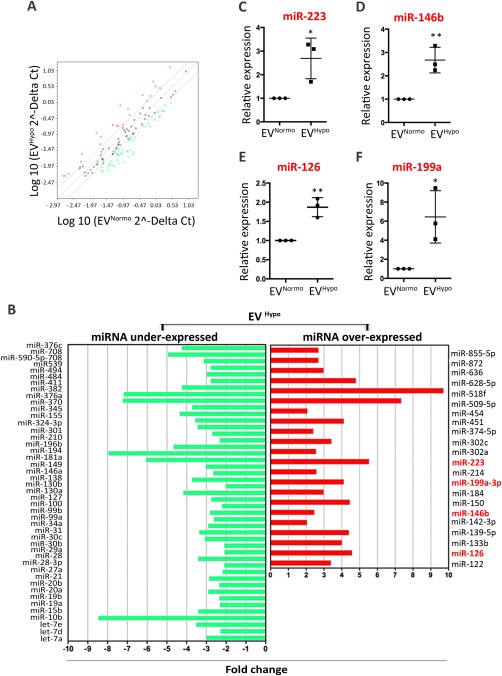
Analysis of miRNAs enriched in EV^Hypo^. **(A)**: Scatter plot of the human miRNome miScript miRNA PCR Array profile depicting the distribution of differentially expressed miRNAs in EV^Hypo^ versus EV^Normo^. The graph was obtained by plotting the normalized log10 miRNA expression (2^−ΔCt^) in EV^Hypo^ (*y*‐axis) divided by the one in the control EV^Normo^ (*x*‐axis). Red and green dots represent upregulated and downregulated miRNAs with an expression level >2 or < 0.5, respectively. **(B)**: Histografic representation of under‐expressed (green) and over‐expressed (red) miRNAs isolated in EV^Hypo^. **(C–F)**: Validation of selected miRNAs expressed by EV^Normo^ and EV^Hypo^ through quantitative real‐time PCR. Normalized data were averaged from three independent experiments, and expressed as mean ± SD. (C): miR‐223, *, *p* = .0274; (D): miR146b, **, *p* = .0062; (E): miR‐126, **, *p* = .0038; (F) miR‐199a, *, *p* = .0264 (unpaired Student's *t* test). Abbreviation: EVs, extracellular vesicles.

miRNAs influence many biological processes and can be taken up as EV cargo also by distant cells 22, 23. To compare the profile of miRNAs present in both EV^Normo^ and EV^Hypo^, each sample was tested for the expression of 384 different miRNAs by PCR array. In order to identify differentially expressed miRNAs in EVs released under hypoxic conditions, raw data were normalized using the small U6 RNA as endogenous reference. Setting EV^Normo^ as control sample and EV^Hypo^ as test sample, the fold change was calculated dividing the normalized gene expression profile of the test sample by the corresponding control sample. The hypoxic cell treatment during the EV release induced the significant over‐expression of 20 miRNAs and the under‐expression of 48 miRNAs (Fig. 2A, 2B). We focused on four specific miRNAs that are implicated in the inflammatory (miR‐223 and miR‐146b) 24, 25, 26, proliferative and differentiative phases (miR‐126 and mir‐199a) 27, 28 of the healing process (Fig. 2C–2F). The significantly upregulated expression of these miRNAs was confirmed by quantitative Real‐Time PCR, thus suggesting that hypoxia‐driven pathways are critical for successful tissue repair.

### MSC‐Derived EVs Promote Macrophage Polarization

In the healing process, macrophages mediate the inflammatory phase by maintaining a pro‐inflammatory phenotype in order to inhibit possible infections. However, they switch to a pro‐resolving, anti‐inflammatory phenotype as soon as the initial “emergency” is over 29, 30.

**Figure 3:**
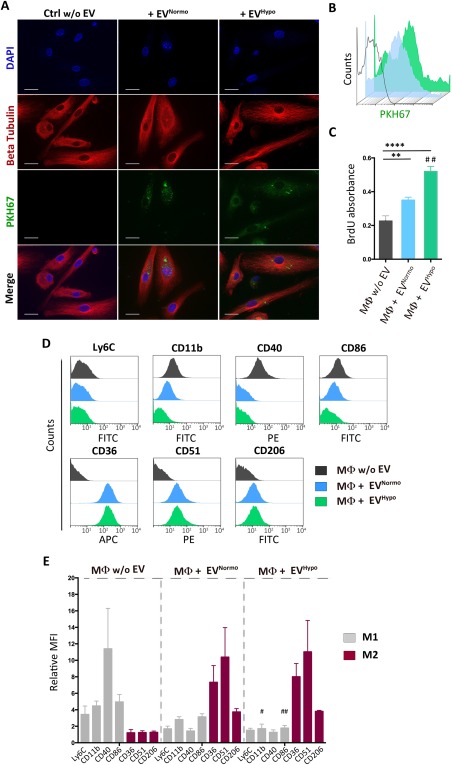
MSC‐derived EVs trigger macrophage (Mϕ) proliferation and polarization. **(A)**: Immunofluorescence analysis of MΦ cocultured for 3 hours in a serum‐free (SF) medium without EVs (Ctrl w/o EV), and with either normoxic or hypoxic PKH67‐labeled EVs (EV^Normo^ and EV^Hypo^). DAPI (blue), β‐tubulin (red), PKH67 (green). Magnification ×63. Scale bar =20 μm. **(B)**: Flow cytometry analysis of MΦ cocultured for 3 hours without EVs (gray line) and with normoxic (light blue line) and hypoxic (green line) PHK67**‐**labeled EVs. **(C)**: Proliferation inductive effect of EV^Normo^ and EV^Hypo^ on MΦ following 24 hour incubation with 10 μM BrdU, compared to the control (MΦ w/o EV). The average of three independent experiments is shown. Statistical significance was determined using ANOVA (**, *p* = .0031; ****, *p* < .0001) and unpaired Student's *t* test to compare EV^Normo^ versus EV^Hypo^ (##, *p* = .0011). **(D)** Representative dot plots of phenotypic characterization of MΦ cultured for 72 hours in SF standard medium (MΦ w/o EV, gray line), and in SF medium containing 1 μg of EV^Normo^ (MΦ + EV^Normo^, light blue line) and EV^Hypo^ (MΦ + EV^Hypo^, green line). Ly6C, CD11b, CD40, and CD86 were selected as markers of pro‐inflammatory MΦ (M1); CD36, CD51, CD206 as markers of anti‐inflammatory MΦ (M2). **(E)**: Histogram presenting the data already shown in panel (D) as relative mean fluorescent intensity. In gray are indicated selected M1 markers and in purple M2 markers. Each column is the average of three independent experiments. Statistical significance was determined using an unpaired Student's *t* test. #, *p* = .0448; ##, *p* =.0095. Abbreviations: DAPI, 4',6‐diamidino‐2‐phenylindole; FITC, Fluorescein isothiocyanate; EVs, extracellular vesicles.

To evaluate the role exerted by EVs in macrophage polarization, we began characterizing the interactions of EVs with recipient cells. We tested whether BM‐derived macrophages (Mϕ) were able to internalize both EV^Normo^ and EV^Hypo^. Mϕ that were incubated for 3 hours in presence of either EV^Normo^ or EV^Hypo^ previously stained with the fluorescent lipophilic membrane‐diffuse dye PKH67, efficiently internalized EVs within their cytoplasm (Fig. 3A). This result was also confirmed by flow cytometry analysis performed after the coculture period on responding cells. More than 70% of EV‐treated macrophages resulted positive for the expression of the FITC‐fluorescent dye PKH67 used to stain EVs and no FITC‐positive signal was detectable in untreated macrophages (Fig. 3B). Cell proliferation of recipient Mϕ maintained for 24 hours in serum free culture conditions was evaluated using a BrdU‐uptake assay. Macrophage proliferation was significantly increased following treatment with both EV^Normo^ and EV^Hypo^ compared to untreated cells (*p* < .0001), and this increase was even greater in hypoxic conditions compared to the normoxic (*p* = .0011) (Fig. 3C).

The flow cytometric analysis of Mϕ maintained in standard culture medium (Mϕ w/o EVs) or in the presence of either EV^Normo^ or EV^Hypo^ was performed. In standard conditions, Mϕ expressed statistically significant higher levels of the pro‐inflammatory M1‐like markers Ly6C, CD11b, CD40, and CD86 compared to the EV‐treated cells (Ly6C: *p* = .0186; CD11b: *p* = .0017; CD40: *p* = .0073; CD86: *p* = .0019) and did not express any of the typical M2 markers, such as the scavenger receptor CD36, the mannose receptor CD206 or the α_v_β_3_ integrin CD51 (Fig. 3D, 3E). Interestingly, 72 hours of treatment with both EV^Normo^ and EV^Hypo^ induced a significant switch of recipient macrophages toward an anti‐inflammatory phenotype (CD206: *p* < .0001; CD51: *p* = .0126; CD36: *p* = .0027) (Fig. 3D, 3E). It is noteworthy that EVs that were released under hypoxic conditions exerted a strengthened anti‐inflammatory effect compared to EVs released under normoxia, downregulating the expression of the co‐stimulatory molecule CD86 and the activation marker CD11b (*p* = .0095 and *p* = .0448, respectively) (Fig. 3E). Taken together, these data indicate that MSC‐derived EVs, and in particular those released under hypoxic conditions, actively interact with key components of the innate immune system and influence their immunoregulatory and regenerative behavior.

### EVs Regulate the M1/M2 Balance of Infiltrating Macrophages in a Skeletal Muscle Injury Model In Vivo

**Figure 4:**
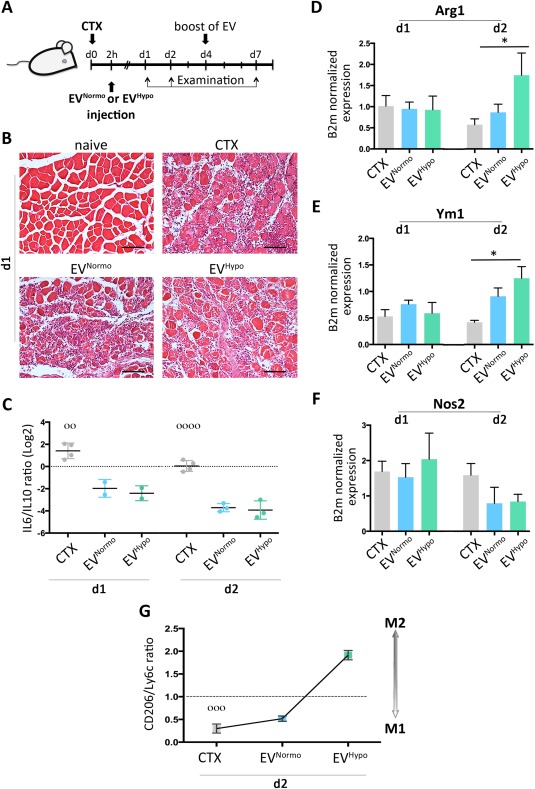
MSC‐derived EVs play direct effects on M1/M2 balance in a mouse model of cardiotoxin‐induced injury. **(A)**: Schematic diagram of the experimental plan, illustrating the timelines of *tibialis anterior* (TA) muscle examination after CTX‐injury and EV‐injection. **(B)**: Representative hematoxylin and eosin (H&E) staining of TA muscles derived from naive C57Bl/6 mouse, CTX‐treated mouse (upper left and upper right panels, respectively), and from EV^Normo^‐ and EV^Hypo^‐treated mice (bottom left and bottom right panels, respectively), one day after damage induction. Magnification ×20. Scale bar = 100 μm. **(C)**: Quantitative real‐time PCR analysis of pro‐inflammatory (IL6) and anti‐inflammatory (IL10) cytokynes in TA muscles derived from mice treated with CTX (gray) alone or in combination with 1 μg EV^Normo^ (light blue) or EV^Hypo^ (green), at 1 and 2 days post‐damage induction. Data are presented as log2 of mean expression ratio ± SEM. Statistical significance was determined using ANOVA. °°, *p* = .0024; °°°°, *p* < .0001. **(D–F)**: Quantitative RT‐PCR for selected M1 (Nos2) and M2 (Arg1, Ym1) markers in CTX (gray), EV^Normo^ (light blue) and EV^Hypo^ (green), at 1 and 2 days post‐CTX injury. B2m has been used as a housekeeping gene. Data are shown as mean ± SEM. Statistical significance was determined using ANOVA and Tukey's multiple comparisons test; (D) *, *p* = .0453. (E) *, *p* = .0087. **(G)**: Flow cytometry analysis of MΦ subtypes recovered in the treated TA muscles (CTX, EV^Normo^ and EV^Hypo^) after 2 days. Data are presented as mean expression ratio between CD206 (M2) and Ly6C (M1). °°°, *p* = .0006 (ANOVA). Abbreviations: Arg1, Arginase 1; CTX, cardiotoxin; EVs, extracellular vesicles; Nos2, nitric oxide synthase 2.

Skeletal muscle has a remarkable capacity for regeneration through a complex injury/repair process that includes inflammation, myofiber regeneration, and angiogenesis 31, 32. Observations that different Mϕ subsets are associated with different stages of muscle regeneration led us to investigate whether EV treatment could influence macrophage polarization from M1 to M2 phenotype in vivo. We opted for a CTX injury in the mouse TA muscle, a reproducible model that recapitulates all healing phases. Muscles, subjected to CTX‐damage followed by injection of either EV^Normo^ or EV^Hypo^, were examined at different times (Fig. 4A). One day after CTX injection, the histopathological evaluation of muscle damage was performed in all experimental groups. Normal myofibers with uniform size, poligonal shape and peripheral nuclei were observed in untreated mice (naive) (Fig. 4B). Following injury, CTX‐treated mice, as well as EV^Normo^ and EV^Hypo^ treated animals, had extensive necrotic muscle fibers with vigorous mononuclear cell infiltrate (Fig. 4B). However, at day 1 and 2 post‐lesion induction, the ratio between *IL6* and *IL10* cytokines (*IL6/IL10*) progressively decreased in EV‐treated muscles compared to CTX‐treated controls (day1: *p* = .0024; day 2: *p* < .0001), thus indicating that the injection of both types of EVs significantly mitigated the inflammatory milieu within the injured tissues (Fig. 4C). At day 2, this observation was accompanied by a significant increase in both types of EV‐treated muscles of the M2 markers Arginase 1 (*Arg1*) and Chitinase 3‐like 3 (*Ym1*) (*p* = .0453 and *p* = .0087, respectively), parallel to a decreased expression of the M1 marker Nitric Oxide Synthase 2 (*Nos2*) (Fig. 4D–4F). The latter results were also confirmed by flow cytometry, analyzing the cells recovered from the damaged and/or EV‐treated muscles. The percentage of CD206‐positive (CD206^pos^) Mϕ compared to the percentage of Ly6C‐positive (Ly6C^pos^) cells was significantly higher within the cells recovered from EV^Hypo^‐treated muscles compared to both CTX‐treated and EV^Normo^‐treated samples (*p* = .0006) (Fig. 4G). Given the important role of Mϕ in muscle regenerative activities, chemokines that are known to attract and interact with these innate immune cells play pivotal roles in the process of muscle recovery after an injury. Among the others, monocyte chemoattractant protein‐1 (MCP‐1) coordinates inflammation‐dependent events involved in muscle regeneration 33. Interestingly, at day 2 post‐lesion induction, the expression level of *MCP‐1* was significantly upregulated in EV^Hypo^‐treated muscles compared to other experimental conditions (*p* = .028) (Supporting Information Fig. 2A). Since CTX‐induced skeletal muscle injury is an optimal model of muscle self‐repair, we analyzed key genes playing a dominant role during the overlapping regeneration and remodeling phases that follow inflammation. At day 7, when compared to CTX‐treated and EV^Normo^‐treated muscles, EV^Hypo^‐treated muscles presented a significant upregulation of both Paired Domain Transcription Factor 7 (*Pax7*) and Myogenic Differentiation Antigen (*MyoD*) genes, selectively expressed by activated satellite cells (*p* = .048 and *p* = .0006, respectively), as well as of embryonic myosin heavy chain (*eMyhc*), expressed by regenerating fibers (*p* = .018) (Supporting Information Fig. 2B). Concurrently, the progression of muscle regeneration and the prospective differences between EV‐treated and CTX‐treated muscles were confirmed by histological observations. As expected, many newly formed centrally nucleated fibers were present in CTX‐treated muscles (Supporting Information Fig. 2C). It's well known that multinucleated muscle fibers form from the fusion of mononucleated myoblasts 34. We observed that the number of mononucleated myoblasts was significantly decreased in both types of EV‐treated muscles, compared to the CTX controls (Supporting Information Fig. 2C–2E, 2G). Interestingly, in the same EV‐treated muscles the number of fibers containing two or more centrally located nuclei was significantly increased compared to the CTX‐injured muscles, and this increase was greater followed EV^Hypo^ injection (Supporting Information Fig. 2C, 2D, 2F, 2G). These results suggest that MSC‐derived EVs, and in particular those released under hypoxic conditions, accelerate the muscle regeneration process.

## Discussion

EVs represent novel players in various cell communication systems, being involved in the regulation of many routes of signaling pathways and intercellular information transfer 35. It is thanks to their vast amount of properties that EVs have been successfully applied in different fields, such as tumor biology, immunology and regenerative medicine 36.

Stem/progenitor cells and in particular MSCs are active biological components of many regenerative medicine therapies 37. Recent efforts in elucidating mechanisms of action of these therapies have revealed an increasingly important role of the cell paracrine activity in enhancing positive outcomes without a significant cell engraftment 13, 38. We recently demonstrated a new role of MSCs in wound healing, showing that they can act as modulators of the inflammatory response, secreting cytokines and factors able to induce the switch of pro‐inflammatory macrophages toward a pro‐resolving, anti‐inflammatory phenotype 10. Indeed, the initial inflammation underlying all regenerative processes is finely coordinated to obtain an efficient outcome, and an altered identity of the inflammatory infiltrate can result in a persistent rather than resolved inflammatory phase 39. Macrophages, that are an essential component of the inflammatory infiltrate, play important roles in the maintenance of tissue homeostasis 40. In response to different signals, macrophages are subjected to a reprogramming and undergo two different polarization states that mirror the Th1/Th2 nomenclature 41. Classical activated M1 macrophages, induced by interferon‐γ alone or in combination with microbial stimuli and/or inflammatory cytokines, exert pro‐inflammatory activities. On the contrary, cytokines such as IL‐4 and IL‐13 induce an alternative activation of M2 macrophages, which become involved in inflammation resolution 42.

Since secreted vesicles represent a relevant component of the MSC regenerative milieu 43, in the present study we investigated the possible role of EVs in modulating the MSC paracrine capacity to actively interact with innate immune cells. Given that the presence of areas of hypoxia is a prominent feature of various inflamed, diseased tissues contributing to modulate the MSC regenerative milieu, these interactions were evaluated after both normoxic and hypoxic cell conditioning 44. We showed that: (a) hypoxic conditioning induced an increased secretion of EVs by MSCs, enriching the EV content in microRNAs involved in different phases of the healing process; (b) MSC‐EVs acted as “switchers” of macrophage polarization toward an anti‐inflammatory phenotype. The latter result was observed both in vitro and in vivo in a mouse model of skeletal muscle regeneration. Literature reports indicate that hypoxia conditioning of MSCs regulates the cargo and protein packaging into EVs 45. As herein shown, the higher expression of both pro‐angiogenic factors and specific microRNAs, such as miR‐223, miR‐146b, miR126, and miR‐199a in response to hypoxia could be at least in part due to a higher number of EVs released by MSCs. Among microRNAs carried by EVs, miR‐223 represents a novel regulator of macrophage polarization, being responsible of suppressing classic pro‐inflammatory pathways and enhancing the alternative anti‐inflammatory responses, whereas the enforced expression of mir‐146b in human monocytes leads to a significant reduction in the production of several pro‐inflammatory cytokines and chemokines, such as IL6 25. In addition, the increased expression pattern of miR‐126 and miR‐199a plays important roles in the repair process restoring vascular integrity and inducing cell differentiation, respectively 27, 28.

An increasing number of literature reports indicate that MSCs possess the capacity to reduce inflammation and to promote tissue repair processes by their paracrine activity 13, 46. In particular, it was recently reported that lipopolysaccharide preconditioning of umbilical cord‐MSCs increased the secretion of exosomes, responsible for the switch of macrophages to a M2‐like profile 47. In line with this evidence, we here demonstrated for the first time that adipose tissue derived‐MSCs release EVs endowed with potent anti‐inflammatory capacities to balance macrophage polarization toward a M2 profile, especially after hypoxic pre‐treatment. The in vitro stimulation of GM‐CSF treated macrophages with either EV^Normo^ or EV^Hypo^ led responding cells to increase their proliferation rate and progressively acquire a M2 phenotype characterized by the expression of CD206, CD51, and CD36. The proper requirement for macrophages is a key feature for efficient muscle regeneration 31, 32. Indeed, macrophages exert specific functions all through the inflammatory response following muscle damage, which includes the sequential release of pro‐inflammatory effectors, the phenotype shift and the activation of myogenic precursors 33. In this context, CTX‐induced skeletal muscle damage represents a highly reproducible model useful to study each step of the inflammatory cascade. The expression level of the typical pro‐inflammatory, Th‐1 cytokine *IL6* was significantly downregulated in EV‐treated muscles at day 1 and 2 post‐lesion induction, that represents the timeframe in which maximum macrophage infiltration occurs 48. This was strictly associated with a significant upregulation of *IL10*, a cytokine that contributes to promote an anti‐inflammatory microenvironment 49. At the same times, the dynamics of macrophage activation marker expression in response to EV administration were investigated. At day 2 the early marker of innate and classical activation *Nos2* was downregulated whereas the expression of *Arg1* and *Ym1*, late markers of alternative activation, were upregulated. This effect was even greater following EV^Hypo^ administration. In the EV‐treated muscles, the changes in the expression of these early/late markers coincided with an increased percentage of CD206^pos^ macrophages. MCP‐1, also known as CCL2, is important in macrophage recruitment and activation. Mice deficient in CCL2/MCP1 show impaired muscle regeneration, characterized by a decrease in the diameter of the new myofibers, a reduced number of capillaries, and fat accumulation 50. In our experimental setting, the administration of hypoxic vesicles determined, at day 2, an accumulation of *MCP‐1* parallel to the macrophage shift toward a M2 phenotype. These concomitant events could underlie the increased expression, at day 7, of the myogenic markers *Pax7* and *MyoD*, that are upregulated and activated by satellite cells, the increased expression of *eMyhc*, that is upregulated by regenerating myofibers, as well as the significantly increased number of newly formed multinucleated muscle fibers, thus indicating an acceleration of tissue repair triggered by EV administration.

When developing novel regenerative medicine strategies, the rational control of inflammation represents a critical aspect to consider. In this context, the anti‐inflammatory, pro‐regenerative effects mediated by MSC‐EVs could be exploited for therapeutic purposes. From a translational perspective, the use of EVs, in comparison to either traditional cell‐based therapies or more recent cell‐free strategies based on the use of MSC secretome, presents undeniable advantages. Compared with traditional cell‐based therapies, the benefits underlying the use of EVs arise in the possibility to develop safer cell‐free therapeutic approaches that could overcome the regulatory obstacles and clinical risks associated to the use of transplanted progenitor cells. Compared to the use of poorly characterized soluble factors, the advantage relies on the ability of EVs to interact and reprogram the surrounding microenvironment, which is a consequence of the variety of their cargo, therefore influencing many biological processes, in particular in injured tissues.

## Conclusion

This study demonstrates that MSCs cultured under both normoxic and hypoxic conditions release EVs endowed with anti‐inflammatory effects. When co‐cultured with responding BM‐derived macrophages, EVs are efficiently internalized by responding cells, inducing, in the short term, an increase in their proliferation rate, and shifting the balance toward a M2 pro‐resolving phenotype. A significant enrichment in microRNAs involved in different phases of the healing process was detectable in EVs especially in the ones derived from hypoxia conditioned MSCs. Direct administration of EVs in a CTX‐induced skeletal muscle injury reduced the inflammatory response, upregulating key markers of alternative activation patterns, and accelerating the expression of myogenic markers. These effects were even greater following EV^Hypo^ administration. Although additional investigations on the mechanisms underlying the therapeutic effects of MSC‐EVs is still necessary before proceeding with clinical trials, these results already provide the basis for the use of EVs as an alternative cell‐free approach for the induction of regenerative processes.

## Author Contributions

C.L.S.: conception and design, collection and/or assembly of data, data analysis and interpretation, manuscript writing; D.R.L.P., C.F., and M.P.: collection and/or assembly of data, data analysis and interpretation; C.B., V.U., E.P., and P.B.: collection and/or assembly of data; M.C.B.: Provision of study material or patients; L.V.: provision of study material or patients, collection and/or assembly of data; R.C.: conception and design, financial support; R.T.: conception and design, financial support, collection and/or assembly of data, data analysis and interpretation, manuscript writing.

## Disclosure of Potential Conflicts of Interest

The authors indicate no potential conflicts of interest.

## Supporting information

Supporting Information Figures.

Supporting Information Table 1.

Supporting Information.
