# Supplementary material for: Mesenchymal Stem Cell‐Derived Extracellular Vesicles as Mediators of Anti‐Inflammatory Effects: Endorsement of Macrophage Polarization
Source: Stem Cells Transl Med. 2017 Jan 31;6(3):1018–28. doi: 10.1002/sctm.16-0363 (PMC5442783; doi:10.1002/sctm.16-0363)
Supplement: Supplementary file 1 — Supporting Information Figures. [file SCT3-6-1018-s001.doc]

**
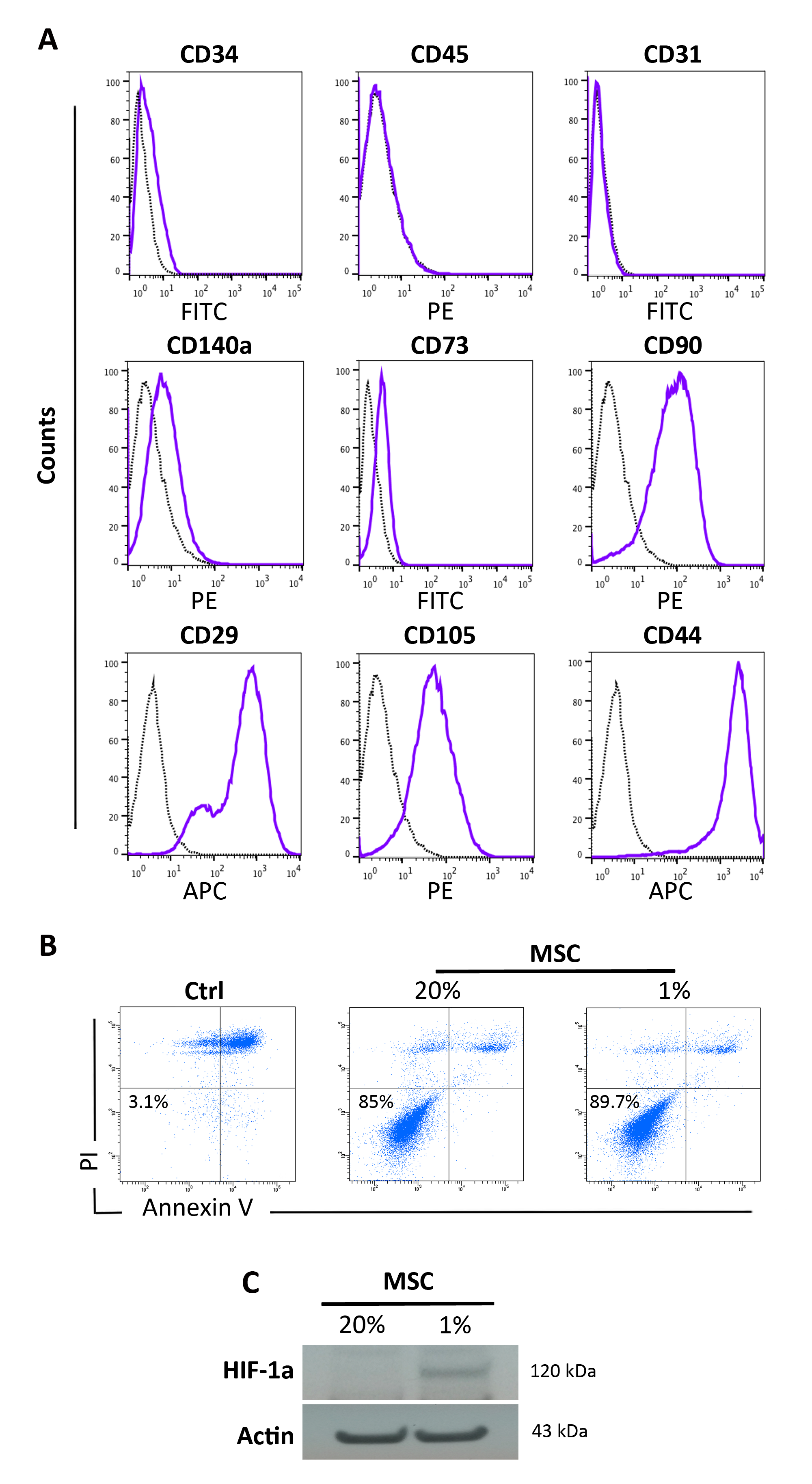
**

**Supplemental Figure S1. Human Adipose tissue-derived Mesenchymal Stem Cells (MSCs) characterization. (A)** Representative flow cytometry analysis of MSCs before being processed for EVs isolation. The purple line represents the expression of the specific marker on tested cell population. The grey line indicates unstained cell suspension, included as negative control. (**B**) Representative flow cytometry analysis of control MSCs (dead cells) and MSCs undergone 48 hour starvation in both normoxic (20%) and hypoxic (1%) culture conditions after staining with Annexin V and PI. (**C**) Representative western blot analysis for HIF-1α expression on MSCs cultured in normoxic and hypoxic condition. Anti-beta Actin was used as an internal control.


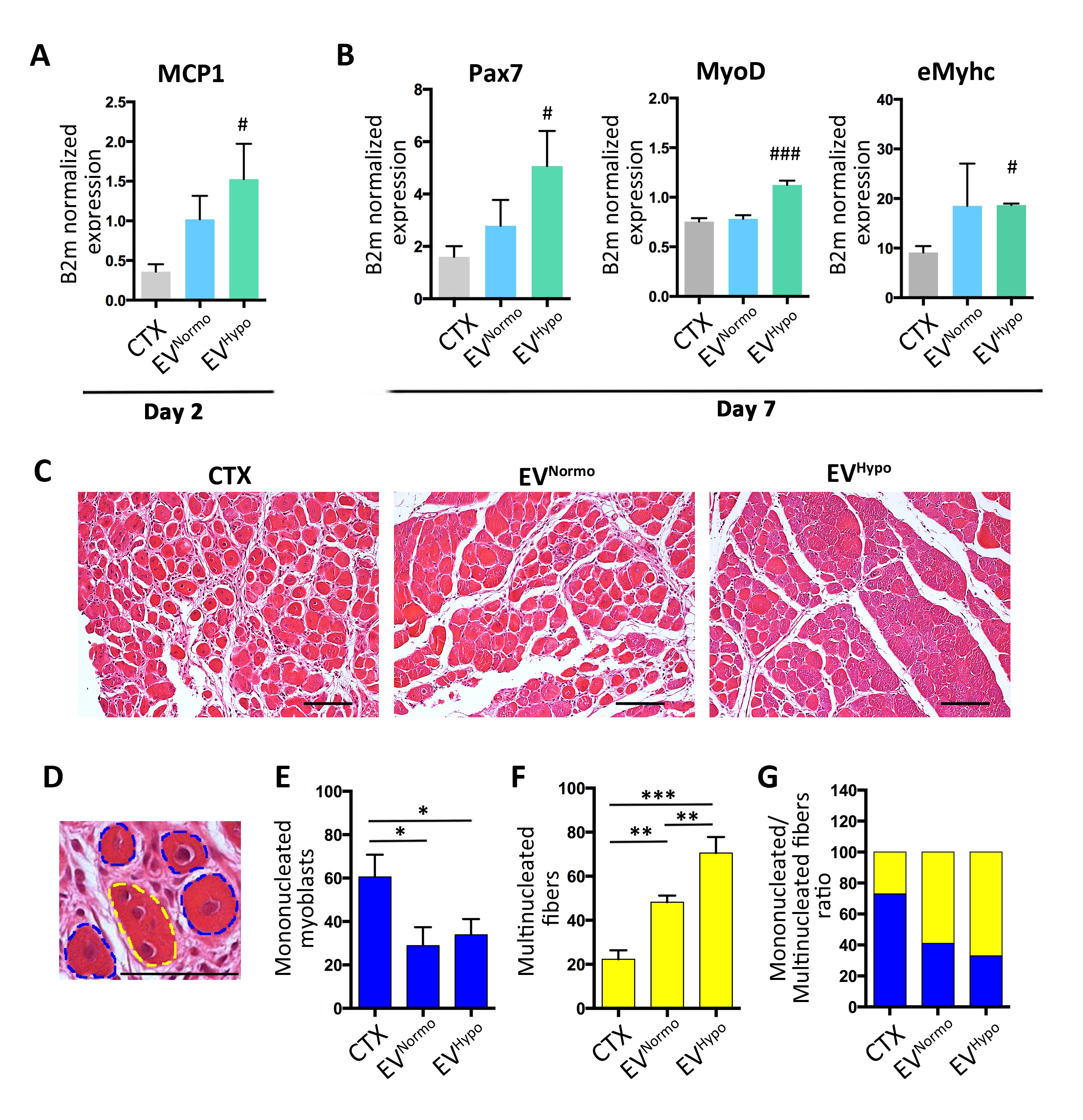


**Supplemental Figure 2. Effect of EV treatment on muscle repair after CTX-damage induction. (A-B)** RT-PCR quantification of myogenic markers expression in TA muscles derived from mice treated with only CTX (grey), and with 1 μg of either EV^Normo^ (light blue) or EV^Hypo^ (green), at 2 and 7 days post CTX-damage induction. B2m has been used as a housekeeping gene. Data are shown as mean ± SEM. Statistical significance of EV^Hypo^ treatment *versus* CTX was determined using an unpaired Student’s t-test (**A**) MCP1= Monocyte Chemoattractant Protein-1, # p=0.028; (**B**) eMyhc = Embryonic Myosin Heavy Chain, # p=0.018. Pax7= Paired Domain Transcription Factor 7, #p=0.048. MyoD = genic Differentiation Protein, ### p= 0.0006. (**C**) Representative H/E staining of *Tibialis Anterior* (TA) muscles derived from CTX-, EV^Normo^- and EV^Hypo^-treated mice, seven days after damage induction. Magnification 20X. Scale bar = 100 μm. (**D**) Representative H/E staining showing the strategy used to count the number of both mononucleated myoblasts, oulined by the blue line, and multinucleated fibers, outlined by the yellow line. (**E-F**) The bar graphs indicate the number of mononucleated myoblasts (**E**) and multinucleated fibers (**F**) present in the TA muscles derived from CTX-, EV^Normo^- and EV^Hypo^-treated mice at day 7 post-lesion induction. Data are shown as mean ± SD. * p= 0.02 (ANOVA) (**E**). ** p≤0.0083; *** p=0.0004 (ANOVA) (**F**). (**G**) 100% Stacked Column Chart indicating the percentages of mononucleated myoblasts (blue portion of the graph) and multinucleated fibers (yellow portion of the graph) in each experimental group.
