# Supplementary material for: Mesenchymal Stem Cell‐Derived Extracellular Vesicles as Mediators of Anti‐Inflammatory Effects: Endorsement of Macrophage Polarization
Source: Stem Cells Transl Med. 2017 Jan 31;6(3):1018–28. doi: 10.1002/sctm.16-0363 (PMC5442783; doi:10.1002/sctm.16-0363)
Supplement: Supplementary file 2 — Supporting Information Table 1. [file SCT3-6-1018-s002.docx]

**Supplemental Tables**

| **Gene** | **FW primer** | **REV primer** |
| --- | --- | --- |
| ***IL-6*** | CTCTGCAAGAGACTTCCATCCAGT | AGTAGGGAAGGCCGTGGTTGTCA |
| ***IL-10*** | CCAGTTTTACCTGGTAGAAGTGATG | TGTCTAGGTCCTGGAGTCCAGCAGACTC |
| ***Nos2*** | GCAGGTCTTTGACGCTCGGA | ATGGCCGACCTGATGTTGCC |
| ***Arg1*** | AGACCACAGTCTGGCAGTTGG | AGGTTGCCCATGCAGATTCCC |
| ***Ym1*** | CTGATCTATGCCTTTGCTGG | AATGTCTTTCTCCACAGACTT |
| ***MCP1*** | GCTCAGCCAGATGCAGTTAACGCCC | CCTTCTTGGGGTCAGCACAGACCT |
| ***eMyhc*** | AGGCCTTGTGCTTTCCCAGAG | GTTCACAGCATGGTGAACCTGG |
| ***Pax7*** | AGCAAGCCCAGACAGGTGGCG | GGCACCGTGCTTCGGTCGCA |
| ***MyoD*** | CGCTCAACTGCTCTTGATG | TAGTAGGCGGTCTCGTAGCC |
| ***B2m*** | GCTTCAGTCGTCAGCATGG | CAGTTCAGTATGTTCGGCTTCC |
| ***Pecam1*** | GGAAGTGTCCTCCCTTGAGC | GCCTTCCGTTCTTAGGGTCG |
| ***VegfA*** | CAAACCTCACCAAAGCCAGC | GCGCTTTCGTTTTTGACCCT |
| ***Hprt*** | CCCCAAAATGGTTAAGGTTGC | CAAACAAAGTCTGGCCTGTAT |

**Supplemental Table 1. Primer sequences.**
