# Supplementary material for: Mesenchymal Stem Cell‐Derived Extracellular Vesicles as Mediators of Anti‐Inflammatory Effects: Endorsement of Macrophage Polarization
Source: Stem Cells Transl Med. 2017 Jan 31;6(3):1018–28. doi: 10.1002/sctm.16-0363 (PMC5442783; doi:10.1002/sctm.16-0363)
Supplement: Supplementary file 3 — Supporting Information. [file SCT3-6-1018-s003.docx]

**Supplemental Materials and Methods**

**Adipose tissue-derived Mesenchymal Stem Cells (MSCs) Isolation and Culture**

Adipose tissue was extensively washed with cold phosphate-buffered saline (PBS: 137 mM NaCl, 2.7 mM KCl, 10 mM Na_2_HPO_4_, 2mM KH_2_PO_4_, pH 7.4) until a clear infranatant solution was obtained. The clean adipose tissue sample was digested in 0.1% type I Collagenase (Gibco, MI, Italy) in PBS at 37°C for 60 minutes. The suspension was then centrifuged at 300 *x*g for 5 minutes at room temperature. After centrifugation, the separation of the stromal cells from the primary adipocytes was completed shaking the samples vigorously to thoroughly disrupt the pellet and mix the cells. After spinning (300 *x*g for 5 minutes) the resulting stromal vascular fraction (SVF) pellet was washed once with PBS, and suspended in Dulbecco's MEM (D-MEM) (Biochrom GmbH, Berlin, Germany) supplemented with 10% fetal bovine serum (FBS) (Lonza, Verviers, Belgium), 2mM of l-glutamine, and 50 mg/mL penicillin/streptomycin. The cultures were performed in the presence of 1 ng/ml of fibroblast growth factor-2 (FGF-2; Peprotech, Milan, Italy) (standard condition). The cells were plated at a density equivalent to approximately 0.2 ml of liposuction tissue aspirate/cm^2^ of surface area, cultured in standard condition with a medium change every 3 days, and expanded after reaching about 85% confluence using 0.05% trypsin-EDTA (Gibco, MI, Italy). All cultures were maintained in a humidified incubator at 37°C with 5% CO_2_. The immunophenotype of cultured MSCs was analyzed by flow cytometry using the following anti-human mABs: CD29 (Clone: TS2/16), CD31 (Clone: WM-59), CD34 (Clone: 4H11), CD44 (Clone: IM7), CD45 (Clone: HI30), CD73 (Clone: AD2), CD90 (Clone: 5E10), CD105 (Clone: SN6) (eBioscience); CD140a (Clone: 16A1) (BioLegend). Only cells that were in passage 2 or 3 were used for extracellular vesicle isolation.

The viability of MSCs after the 48-hour starvation period in both normoxic and hypoxic conditions was evaluated by flow cytometry using the FITC Annexin V Apoptosis Detection Kit I (BD Biosciences).

**Bone Marrow (BM)-derived Macrophage Isolation and Culture**

Bone marrow nucleated cells were washed in alpha minimum essential medium (α-MEM) and plated in a 10-mm-diameter cell culture dish. After 2 h, the supernatant containing the cells that did not adhere to the dish was collected and plated in a 150-mm "non-treated" culture dish (Iwaki), and α-MEM supplemented with 10% FBS, 2mM l-glutamine, 50 mg/mL penicillin/streptomycin, and granulocyte macrophage–colony stimulating factor (GM-CSF; 25 mg/mL) was added (complete α-MEM).

**Transmission Electron Microscopy (TEM)**

For MSC analysis, the cells were fixed with 2.5% glutaraldehyde (Fluka, St. Louis, MO, USA) in 0.1 M phosphate buffer (PB) pH 7.3, for 1h at 4°C. The pellet was subsequently washed twice in PB, post-fixed in 2% osmium tetroxide, dehydrated in a graded series of ethanol up to absolute, pre-infiltrated, and embedded in Epoxy resin (Epon 812). Ultrathin sections (90 nm) were mounted on 200-mesh copper grids, stained with uranyl acetate and lead citrate. For EV analysis, about 20 µl of vesicle suspensions were placed on Parafilm (Bemis, Neenah, WI, USA). Formvar-coated copper grids (Electron Microscopy Sciences, Hatfield, PA, USA) were placed over them, in a moist chamber, for 1 h at room temperature. Grids were then briefly washed in 0.1 M PB, pH 7.3, fixed for 10 min with 2.5% glutaraldehyde and contrasted with 2% uranyl acetate (Electron Microscopy Sciences). Both EVs and MSCs were finally observed under a Philips EM 208 microscope equipped with a digital camera (University Centre for Electron Microscopy, CUME, Perugia, Italy).

**Protein Quantification and Immunoblot Analysis**

EVs (n=6 for both conditions) were prepared by homogenization in SDS buffer and collected from five independent experiments. Electrophoresis was performed in reducing conditions on a 4%–12% NuPAGE Bis-Tris gel (Invitrogen, MI, Italy) as previously described [18]. For each sample, 3 μg of EV proteins was separated on the gel and transferred to a Protran BA83 nitrocellulose membrane (Whatman GmbH, Dassel, Germany). The blot was incubated with specific primary antibodies and with anti-mouse secondary antibodies, revealed with an enhanced chemo luminescence substrate mixture (Amersham), and exposed to an X-ray film (Amersham). Images were then scanned using the Epson perfection 1260 scanner (Epson, Italia). Antibodies anti-CD81, and anti-Alix were supplied by Santa Cruz Biotechnology (Dallas, TX). Antibody anti-beta Tubulin was supplied by AbCam. MSC^Normo^ and MSC^Hypo^ lysates were collected for protein concentration determination using the Bradford assay and for HIF-1α (clone 54, BD Bioscience) expression analysis.

**In vivo Angiogenic Assay**

Three-hundreds μl of unpolymerized liquid matrigel (Basement membrane Matrix, Corning) were injected subcutaneously into the flanks of 8- to 12-week-old C57/Bl6 male mice under general gas anesthesia (2% isoflurane concentration) in combination with: i) 5μg of EV^Normo^ suspended in 100 μl PBS; ii) 5μg of EV^Hypo^ suspended in 100 μl PBS; iii) 100 μl PBS (negative control). Groups of 3 mice were used for each treatment. Three weeks after injection, matrigel plugs were recovered and processed. Each plug was divided into 2 parts in order to perform both histological (see " Histology and Immunofluorescence" paragraph) and PCR analysis. Plugs were homogenized in Qiazol Lysis Reagent (Qiagen) and total RNA was extracted by RNeasy plus mini kit (Qiagen), quantified by optical density (OD) measurement, and checked for quality. cDNA synthesis was performed using Omniscript Reverse Transcription Kit (Qiagen). PCR reaction was performed using specific primers listed in Supplemental Table 1 (Thermo Scientific 2X Reddy Mix PCR Master Mix AB-0575/DC/LD/A). PCR products were analyzed by densitometry using ImageJ software.

**Flow cytometry Analysis**

Using a reverse pipetting technique, 50 μl of diluted stained sample of EVs were pipetted into a TruCount tube (BD Biosciences) to which 300 μl of binding buffer were then added. TruCount tubes, containing a standardized number of fluorescent beads, were used to quantify EVs. Sample acquisition was discontinued when the number of TruCount beads in the relative region reached 10,000 events. The number of EVs per microliter was normalized to the number of MSC^Normo^ and MSC^Hypo^ (1x10^6^) and was calculated as indicated in TruCount data sheet.

The immunophenotype of Mφ cultured for 72 hours in the presence of either EV^Normo^ or EV^Hypo^ (1μg/1x10^4^ cells) was compared to that one maintained in standard culture medium (Mφ w/o EVs) by flow cytometry, using the following antibodies anti-mouse mAbs: CD11b (M1/70), CD51 (RMV-7), CD86 (GL1) (BD Pharmingen), CD36 (No.72-1), CD40 (1C10) (eBioscience), Ly6C (1G7.G10) (Miltenyi Biotech) and CD206 (MR5D3) (BioLegend).

The immunophenotype of macrophages infiltrating the injured *tibialis anterior* (TA) muscles was also analyzed. Two days after cardiotoxin (CTX)-induced injury, muscle treated with CTX alone or in combination with EV^Normo^/EV^Hypo^ were harvested, minced, and enzymatically digested with 1 mg/ml Collagenase/Dispase solution in PBS (Sigma-Aldrich) at 37°C for 90 minutes. The obtained cell suspension was analyzed by flow cytometry as previously described.

All flow cytometry analyses reported in this study were performed using the BD FACSAria II. Acquisition and analysis of data were performed by FACSDiva software (BD Biosciences) or FlowJo software. Data are expressed as percentages of positive cells or as ratio between mean fluorescence intensity (MFI) of cells stained with a specific antibody/MFI of correspondent isotype control (relative MFI).

**RNA Extraction**

The EV pellet was suspended in 500 μl of QIAzol lysis reagent (Qiagen) and stored at -20°C. Total RNA derived from EV pellet was extracted using the combination method TRIzol/RNeasy. Samples (n=3) were thawed, homogenized and chloroform was added at 1:5 ratio. Samples were centrifuged at 13000 rpm for 15 minutes and the upper aqueous phase containing RNA was collected. After adding 1.5 volumes 100% ethanol, total RNA was purified using RNeasy Mini kit (Qiagen) according to the manufacturer's recommendations. Quantification and quality assessment of small RNA including the miRNA fraction was performed with the 2100 Bioanalyzer using the Small RNA kit (Agilent Technologies, Waldbronn, Germany) according to manufacturer’s instructions. Small RNA analyses were carried out using the Agilent 2100 expert software.

The RNA extraction from TA muscles was performed by TissueLyser (Qiagen) homogenization along with TRizol for 5 minutes at 30 Hz, according to the manufacturer's recommendations.

**microRNA Profiling**

Fifty ng of the total RNA were reverse transcribed using the Megaplex RT Primers Human Pool A. At the end of the reaction, each RT product was pre-amplified with the Megaplex PreAmp Primers A for 12 cycles. Then, the pre-amplification products were loaded onto MicroRNA TaqMan Card A (v 2.0). Card amplifications were performed on ViiA7 equipment for 40 cycles. All reagents and equipment were from Life Technologies. Results were expressed as delta Cq by subtracting the Cq value obtained for U6 small RNA from the Cq value of each miRNA. For unexpressed miRNAs the Cq value was set at 40.

**Quantitative Real-Time PCR**

Ten ng EV RNA was reverse transcribed by the miScript II RT kit (Qiagen) at 37°C for 60 min, and then the enzyme was inactivated at 95°C for 5 min. After the activation of the polymerase enzyme at 95°C for 15 min, 40 cycles of 94°C for 15 s, 55°C for 30 s, and 72°C for 30 s were performed. Melting curve analysis was used to confirm the specificity of the amplification reactions. Gene expression levels were normalized to the corresponding miRNAs in MSC by applying the 2^-ddCt^ method.

For the quantification of *IL-6, IL-10, Nos2, Arg1, Ym1, MCP1, eMyhc, Pax7,* and *MyoD* mRNAs in TA muscles of CTX and EVs injected mice, total RNA was retro-transcribed with SuperScript II and related products (all from Life Technologies) in a 20 ml reaction. Real-time PCR reactions were performed using a LightCycler II (Roche, Monza, Italy). Reactions were carried out in triplicate using 4 ml of FASTSTART SYBR GREEN MASTER (Roche) and 2 ml of primers mix FW. REV (final concentration, 300/300 nM) in a final volume of 20 ml. Briefly, serial dilutions of a positive control sample were used to create a standard curve for the relative quantification. The amount of each mRNA was normalized for the content in β2-microglobulin [18]. Primer sequences are listed in Supplemental Table 1.

**Immunofluorescence analysis**

Mφ were prepared for fluorescence microscopy by permeabilization for 5 minutes with 0.1% Triton-X100, blocked with 5% BSA and incubated with anti-beta Tubulin antibody (Abcam, Cambridge, UK) for 2 hours. Cells were incubated with secondary antibody, Alexa Fluor 594-conjugated goat anti-rabbit IgG antibody (Molecular Probes; Invitrogen) for 1 h at room temperature. A DAPI solution (Abbott Molecular Inc, Des Plaines, IL, USA) was applied for 5 minutes for nuclear staining. Images were captured using an Axiovert 200 M microscope (Zeiss, Germany).

**Histology and morphometric analysis**

At 1 and 7 days post-administration of CTX (n=2) with or without 1μg of either EV^Normo^ or EV^Hypo^ (n=3 for each treatment), mice were sacrificed and the TA muscles were collected and placed in 3.7% paraformaldehyde for 2h before routine paraffin embedding. For morphometric analysis, 4μm sections of transversely cut muscles fibers were deparaffinized and stained with H/E. The number of mononucleated myoblasts and the number of fibers containing two or more centrally located nuclei were determined by counting individual myofibers in digitized images of a given TA muscle by using ImageJ Tool. Within each experimental group, six non-overlapping areas derived from all the TA-treated muscles were digitally captured (20X magnification) by transmitted light microscopy with an Olympus C3030 digital camera. Results were expressed as number of fibers in a fixed region of interest (ROI) of 210 mm^2^.
